# Viral Evolution and Cytotoxic T Cell Restricted Selection in Acute Infant HIV-1 Infection

**DOI:** 10.1038/srep29536

**Published:** 2016-07-12

**Authors:** Miguel A. Garcia-Knight, Jennifer Slyker, Barbara Lohman Payne, Sergei L. Kosakovsky Pond, Thushan I. de Silva, Bhavna Chohan, Brian Khasimwa, Dorothy Mbori-Ngacha, Grace John-Stewart, Sarah L. Rowland-Jones, Joakim Esbjörnsson

**Affiliations:** 1 Nuffield Department of Clinical Medicine, University of Oxford, Oxford, United Kingdom; 2 KEMRI-Wellcome Trust Research Program, Centre for Geographical Medicine Research, Kilifi, Kenya; 3 Department of Global Health, University of Washington, Seattle, Washington, United States of America; 4 Department of Paediatrics and Child Health, University of Nairobi, Nairobi, Kenya; 5 Department of Medicine, University of Washington, Seattle, Washington, United States of America; 6 Department of Medicine, University of California San Diego, San Diego, California, United States of America; 7 Section of Paediatrics, Department of Medicine, Wright Fleming Institute, Imperial College London, London, United Kingdom; 8 Kenyan Medical Research Institute, Nairobi, Kenya; 9 Department of Epidemiology, University of Washington, Seattle, Washington, United States of America; 10 Department of Pediatrics, University of Washington, Seattle, Washington, United States of America; 11 Department of Microbiology, Tumor and Cell Biology, Karolinska Institute, Stockholm, Sweden

## Abstract

Antiretroviral therapy-naive HIV-1 infected infants experience poor viral containment and rapid disease progression compared to adults. Viral factors (e.g. transmitted cytotoxic T- lymphocyte (CTL) escape mutations) or infant factors (e.g. reduced CTL functional capacity) may explain this observation. We assessed CTL functionality by analysing selection in CTL-targeted HIV-1 epitopes following perinatal infection. HIV-1 *gag*, *pol* and *nef* sequences were generated from a historical repository of longitudinal specimens from 19 vertically infected infants. Evolutionary rate and selection were estimated for each gene and in CTL-restricted and non-restricted epitopes. Evolutionary rate was higher in *nef* and *gag* vs. *pol*, and lower in infants with non-severe immunosuppression vs. severe immunosuppression across *gag* and *nef*. Selection pressure was stronger in infants with non-severe immunosuppression vs. severe immunosuppression across *gag*. The analysis also showed that infants with non-severe immunosuppression had stronger selection in CTL-restricted vs. non-restricted epitopes in *gag* and *nef*. Evidence of stronger CTL selection was absent in infants with severe immunosuppression. These data indicate that infant CTLs can exert selection pressure on *gag* and *nef* epitopes in early infection and that stronger selection across CTL epitopes is associated with favourable clinical outcomes. These results have implications for the development of paediatric HIV-1 vaccines.

Clear differences in the natural history of acute infection exist between adults and infants both clinically and with respect to viral population dynamics and the nature of the cytotoxic T cell (CTL) response. Where adults typically progress to AIDS over 8–10 years if untreated, infants experience extremely rapid progression, with 50% of African infants dying before they are 2 years old^1^,^2^. Despite the uncontrolled viral replication and high mortality rates associated with vertical HIV-1 transmission, a proportion of infants experience delayed clinical and immunological progression in the absence of antiretroviral therapy that can persist into childhood and even adolescence^3^. The mediators of infant immunity that contribute to slow progression in paediatric HIV-1 infection remain largely unknown.

Acute HIV-1 infection in adults is characterised by a steep decline from peak viral load during week 12–20 post-infection to a stabilised viral load set point that has been positively correlated with the rate of disease progression^4^,^5^. This decline and set point maintenance is largely mediated by HIV-1 specific CTL responses. Vertically infected infants experience a higher peak viraemia compared to adults^6^, a slower decline in viral load over the first two years of infection^7^ and lack a well-defined viral load set point. However, albeit on a lower magnitude compared to adults, vertically infected infants can mount HIV-1 specific CTL responses from birth^8^,^9^,^10^. This indicates that infant HIV-1 specific responses can be functional and select for viral escape variants^11^,^12^.

The rate of evolution in HIV-1 is one of the highest in nature, and a striking degree of viral genetic diversity is observed within HIV-1-infected individuals^13^. The error prone nature of the viral reverse transcriptase, a high rate of recombination, and a high replicative capacity all contribute to the genetic diversity. This plasticity enables viral adaptation to the host immune response^14^ and the functional efficacy of the CTL response can be observed by the selection of viral escape variants and differences in CTL-restricted epitopes^15^,^16^,^17^. Detailed longitudinal characterisation of the HIV-1 specific immune response has shown that CTLs exert a significant selective pressure on HIV-1 in early adult infection^15^,^18^. However, it is not known to what extent CTL-mediated selection pressure drives HIV-1 evolution in early life of infants, and how these functional differences may determine viral control and ultimate clinical outcome. Selection pressure such as that imposed by the host CTL response will influence the evolutionary rate of HIV-1 and the ratio of nonsynonymous and synonymous substitutions (d*N*/d*S*) provides a robust measure of both the mode and strength of selection. The intra-host evolutionary dynamics of HIV-1 and its relationship to clinical outcome has been studied in both adults^19^,^20^,^21^,^22^ and infants^23^,^24^,^25^,^26^, with the majority of studies focusing on the envelope gene encoding the primary targets of the humoral immune response^27^. The major targets of the CTL response, however, lie principally in the *gag, pol* and *nef* genes. Although the relationship between the evolutionary dynamics in these genes and clinical outcomes has been explored to some extent in adults^28^,^29^, little is known about this relationship during paediatric HIV-1 infection.

In a cohort of perinatally infected Kenyan infants, we estimated joint and individual evolutionary rates and selection across *gag*, *pol* and *nef*. We also investigated the influence of the infant CTL response on evolutionary parameters. Finally we assessed the association between clinical outcome and selection in CTL epitopes. Our results indicate a clear association between stronger immune selection on CTL-restricted epitopes and less severe immunosuppression during early infant HIV-1 infection.

## Results

### Participant characteristics

HIV-1 *gag*, *pol* or *nef* sequences at three or more longitudinal time points were recovered from 19 of 23 eligible infants (Table 1). From 83 available specimens sequentially collected during the first 24 months of infant HIV-1 infection, sequence recovery was 98% for *gag*, 64% for *pol* and 87% for *nef*. Of the 19 infants, 13 (68%) were male, 12 (63%) became infected *peripartum* and seven (37%) became infected *in utero*. The median peak viral load was 7.2 log_10_ viral RNA copies/mL of plasma (interquartile range [IQR]: 6.6–7.6); the median absolute CD4 T cell count and CD4% at 6 months of age were 1415 cells/mL (IQR: 760–2195 cells/mL) and 22% (IQR: 16–26%), respectively (S1 Table). As expected, phylogenetic analysis showed clustering of sequences sampled longitudinally for the same infant (Fig. S1). The baseline characteristics of the 19 infants were representative of infants in the parent cohort (S1 Table). Subtype analysis showed that 11 infants were infected with HIV-1 sub-subtype A1 (58%) and one infant with HIV-1 subtype D (5%). This result was concordant in all the analysed genetic regions. Seven infants (37%) showed discordant subtype classification between HIV-1 genes that was unique from previously described circulating recombinant forms (CRFs), indicating a large proportion of unique recombinant forms (URFs, S2 Fig.).

### HIV-1 evolutionary rates were highest in *nef* followed by *gag* and *pol*

**Figure 1:**
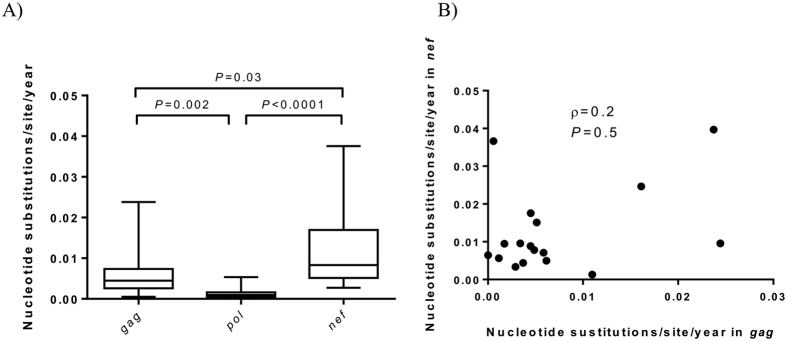
Evolutionary rates across HIV-1 *gag, pol* and *nef* genes from infants sampled under 2 years of age. (**A**) Summary of nucleotide substitution rates across *gag, pol* and *nef* with median (line) 1^st^ and 3^rd^ quartiles (box) and the 10^th^ and 90^th^ percentile (whiskers) shown. The Mann Whitney U test was used to assess differences between groups. (**B**) Rank-based Spearman correlations between the nucleotide substitution rate across *gag* and *nef*.

The average evolutionary rates for *gag, pol*, and *nef* were 5.9 (95% confidence intervals [CI]: 5.0–7.0), 1.5 (CI: 1.0–2.0) and 12.6 (CI: 10.0–15.0) nucleotide substitutions/1000 sites/year, respectively. The average evolutionary rates were significantly higher in *gag* and *nef* compared to *pol* (*P* = 0.002 and *P* < 0.0001, respectively), and in *nef* compared to *gag* (*P* = 0.03, Fig. 1A). The patient-specific evolutionary rates across *gag* and *nef* did not correlate (ρ = 0.2, *P* = 0.5; Fig. 1B) and across *pol* it was consistently low between individuals, indicating considerable evolutionary constraint. Overall, d*N*/d*S* ratios indicated moderate purifying selection across *gag* and *nef* (0.7 [CI: 0.5–0.8] and 0.8 [0.6–0.9], respectively), and strong purifying selection across *pol* (0.3 [CI: 0.2–0.4]). Individual estimations indicated that four (IDs: 170, 211, 291 and 334), zero and one (ID: 211) infants showed overall positive selection across *gag*, *pol* and *nef*, respectively.

### Evolutionary rate was lower in infants with non-severe immunosuppression than in infants with severe immunosuppression across *gag* and *nef*

In order to assess associations between clinical outcomes and evolutionary parameters, infants were stratified into groups based on severe immunosuppression (nadir CD4% ≤15), peak viral load, mode of transmission, and HIV-1 subtype (Table 2). The evolutionary rate was significantly lower in infants with non-severe immunosuppression compared to those with severe immunosuppression across *gag* and *nef* (*P* = 0.008 and 0.008, respectively; Table 2). In addition, selection was significantly stronger in infants with non-severe compared to those with severe immunosuppression across *gag* (1.4 [CI: 1.1–1.8] vs. 0.4 [CI: 0.3–0.5]; *P* = 0.006). Three of four infants with overall positive selection across *gag* (IDs: 170, 211 and 334), and the single infant with global positive selection across *nef* (ID: 211) were all infants with non-severe immunosuppression. No significant difference was observed in median peak viral load between non-severely immunosuppressed and severely immunosupressed infants (6.6 vs 7.3 log_10_ viral RNA copies/mL of plasma, *P* = 0.2 [Mann-Whitney U test]).

The evolutionary rate in *nef* was higher in infants with high peak viral loads (above median peak viral load) compared to those with low peak viral loads (below median, *P* = 0.007). The evolutionary rate was also higher in *nef* in infants infected with HIV-1 non-A subtypes compared to those infected with HIV-1 subtype A (*P* = 0.018). Interestingly, five of seven non-subtype A infected infants were infected with URFs, and all these five infants had *nef* sequences that clustered with subtype A-like sequences. No significant differences were observed between evolutionary rate estimates and selection across *pol* between infant groups (Table 2), or between evolutionary parameters in any genetic region in infants stratified by mode of transmission.

### CTL targeted regions were under stronger selection than non-CTL targeted regions in *gag* and *nef*

To investigate the influence of infant CTL responses in early viral evolution, we used infant HLA types to partition viral sequences into epitopes putatively restricted by the infant CTL response (CTL+) and non-restricted parts (CTL−). Differences in estimates of evolutionary rate and selection strength was assessed on both the group level and the individual level. Overall, no significant differences in evolutionary rate between CTL+ and CTL− regions was seen across *gag* and *pol*. However, on the individual level, three infants (IDs: 261, 313 and 334) had significantly higher evolutionary rate in CTL+ vs. CTL− regions. (P < 0.05 for all comparisons). Overall, the *nef* gene showed contrasting results with significantly higher evolutionary rate in CTL+ regions (18.4 nucleotide substitutions/1000 sites/year [CI: 14.0–24.0]) compared to CTL− regions (9.1 [CI: 7.0–12.0], P < 0.001). Individual comparisons indicated faster rates in CTL+ regions in five infants (IDs: 170, 211, 231, 281, 411 and 454) and slower rates in one infant (ID: 424) compared to CTL− regions (*P* ≤ 0.05 for all comparisons).

Estimates of selection in partitioned genetic regions indicated that d*N*/d*S* ratios in *gag* were significantly higher in CTL+ (1.0 [CI: 0.7–1.4]) vs. CTL− regions (0.5 [CI: 0.4–0.7], *P* = 0.008). Individual estimates indicated that the same three individuals with faster evolutionary rates in CTL+ vs. CTL− regions, also had significantly stronger selection in CTL+ vs. CTL− regions (P < 0.05 for all comparisons). Similar results were seen in *nef* with significantly higher d*N*/d*S* ratios in CTL+ (1.2 [CI: 0.8–1.6]) vs. CTL− regions (0.5 [CI: 0.4–0.7]; *P* = 0.001) on the group level. Individual estimates indicated that six infants had significantly stronger selection in CTL+ vs. CTL− regions (P < 0.05 for all comparisons), four of which (IDs: 170, 211, 231, and 411) were also the infants with higher evolutionary rates in CTL+ vs. CTL− regions. No significant differences in selection pressure between CTL+/− regions were observed in *pol* at either the group level or the individual level.

### CTL targeted regions across *gag* and *nef* were under stronger selection in infants with non-severe immunosuppression in contrast to infants with severe immunosuppression

To further explore the relationship between CTL-mediated selection in *gag* and *nef* and the clinical outcome of infection, we estimated differences in d*N*/d*S* ratios in CTL+/− regions in infant groups stratified by the severity of immunosuppression. The *pol* region was not considered due to the consistently low variation and evolutionary signal. The d*N*/d*S* ratios were higher in CTL+ vs. CTL− regions in infants with non-severe immunosuppression in both *gag* (2.3 [CI: 1.6–3.3] vs. 1.0 [CI: 0.7–1.5], *P* = 0.006) and *nef* (2.6 [CI: 1.7–3.8] vs. 0.5 [CI: 0.2–0.8], *P* < 0.001; Table 3). No significant differences were found between d*N*/d*S* ratios across CTL+ and CTL− regions in infants with severe immunosuppression in either *gag* or *nef*.

## Discussion

The positive correlation between HIV-1 evolutionary rate and disease progression rate has been demonstrated several times in adults^20^,^22^,^30^. Here, we show that this is true in infant HIV-1 infection as well. We also demonstrate a clear association between stronger immune selection on CTL-restricted epitopes and less severe immunosuppression during early infant HIV-1 infection. However, in contrast to adults, evidence of CTL-mediated selection is only seen in a minority of vertically-infected infants who maintain relatively high CD4 percentages. Our results support the notion that infant CTL responses may have the functional capacity to exert selective pressure on cognate epitopes, and that possession of functional CTL responses is associated with favourable clinical outcomes. The strengths of our study approach include the use of historical samples obtained before the era of infant ART, a clinically well characterised cohort, a relatively large sample size which enabled stratification of individuals according to clinical parameters, and the analysis of longitudinal changes in viral sequence in early infant HIV-1 infection.

The few studies that have addressed the association between CTL-mediated evolution and differential clinical outcomes in infant HIV-1 infection have reported divergent findings. Recently, Adland *et al*.^31^ indicated that HLA played a limited role in protection from HIV-1 disease progression in children compared to adults, with modest associations between the presence of a protective HLA allele and disease severity in children or with *in vitro* viral replicative capacity of patient derived chimeric viruses. In addition, no evidence of *de novo* CTL escape was seen in viral sequences from severely immunosuppressed infants (CD4% <20 within the first year of life) bearing protective HLA B alleles. Our data indicate that CTL-mediated selection indeed can occur as a consequence of the infant CTL response and that this is associated with non-severe immunosuppression. This suggests a possible protective role for HLA in mediating HIV-1 disease outcomes in early infection following vertical transmission, in some but not all infants, potentially explaining the inconsistent findings in previous reports. Our results are in accordance with a study of five infants with no evidence of severe immune suppression (based on the CDC paediatric HIV-1 classification), which reported global positive selection in *nef* and CD8 T cell driven mutations in HLA A*24-restricted *nef* epitopes over the first year of life^11^. Similarly, amino acid changes in CTL-associated *nef* epitopes was reported for children with non-progression but not for children with disease progression^32^. As discussed by Adland *et al*.^33^, further evidence of functional immune driven selection in neonatal HIV-1 infection comes from observations that replicative capacity of infant Gag-Protease chimeric viruses was significantly reduced compared with matched maternal chimeric viruses^34^. Our data suggest that within the first year of life, a proportion of infants may select viral variants that harbour CTL-driven mutations. Further studies are needed to determine associations between CTL-driven mutations, fitness costs and viral load dynamics in early infant infection.

Despite the apparent association between CTL-driven selection in HIV-1 genes and clinical outcome in this study, uncontrolled viremia was observed throughout the first two years of life in the majority of infants. Some of the unique features of paediatric immunity may account for this discrepancy, particularly with regards to the increased CD4 T cell counts that are seen in infancy both in the periphery and in mucosa when compared to adulthood. Although naïve (CCR5^−^) CD4 T cells predominate in the periphery, evidence from the rhesus macaque model indicate that the pool of activated and proliferating CD4^+^ targets is greatly increased in neonatal tissues compared to adults (10-fold in the gut), and that these cells are preferentially targeted and depleted by simian immunodeficiency virus^35^,^36^. The abundance^37^ and profound loss of CCR5^+^ CD4 coupled with the high rate of thymic turnover during infancy, may supply HIV-1 with a virtually unlimited supply of susceptible target cells. As a consequence, functional infant CTL responses may not reflect the dynamics of peripheral blood viraemia in early life because these are masked by rapid viral turnover in the context of overabundance of susceptible target cells. Notwithstanding, we observed a large effect size of the median difference between peak viral loads in non-severely immunosuppressed vs severely immunosuppressed groups that did not reach significance, likely due to a power effect. This suggests a relationship between infant pathogenesis, peak viral load and evolutionary dynamics that merit further studies.

Due to the use of viral population sequences, our estimates of evolutionary rate across *gag, pol* and *nef* probably provide a lower bound on this parameter^17^. Our estimates ranged from 10^−2^ nucleotide substitutions/site/year in *nef*, to 10^−3^ nucleotide substitutions/site/year in *gag* and *pol* which is in accordance with rates estimated during adult infection^27^,^38^, although direct comparisons with previous reports may be difficult to interpret due to methodological differences. Our analysis was adapted from Hightower *et al*. who reported an average rate of 0.72 nucleotide substitutions/1000 sites/year across *pol* during adult chronic HIV-1 subtype B infection^17^. Our estimate of 1.5 nucleotide substitutions/1000 sites/year in *pol* is approximately twice as high and could reflect differences in evolutionary rate between early and chronic infection^19^ or differences between adult and infant infection (e.g. higher viral loads in infants that may influence evolutionary rates). However, the low evolutionary rate across *pol* relative to *gag* and *nef* suggests considerable evolutionary constraints in this region, as would be expected from genes encoding enzymes with critical functions in the viral replication cycle.

In accordance with previous estimates^27^, *nef* was shown to evolve at the highest rate, a reflection of the molecular plasticity of its encoded protein that is involved in diverse roles during viral replication and pathogenesis. In addition, the influence of CTL selection was most apparent in *nef* as a) six infants showed significantly stronger selection in CTL+ vs. CTL− regions compared to three across *gag*, and b) the overall association between CTL selection and favourable clinical outcome resulted in higher LRT scores in *nef* compared to *gag*. This observation probably reflects the early targeting of *nef* by CTL responses as seen in both adult HIV-1 infection and in SIV infection. Comparably, CTL targeting of *gag* epitopes has been shown to occur at a later stage of infection. Alternatively, these observed differences may reflect strong protective effects of CTL selection in *nef* during acute HIV-1 infection.

We also found a lower evolutionary rate in *nef* among HIV-1 subtype A compared with non-subtype A infected infants. This result together with the higher viral evolutionary rate seen in infants with severe immunosuppression are in line with previous studies on adults that show a positive correlation between rate of evolution and disease progression^20^,^22^,^30^ and a slower disease progression in HIV-1 subtype A-infected adults compared with other HIV-1 subtypes and CRFs^39^,^40^. These results warrants further studies on HIV-1 strain-specific effects on disease progression among both infants and adults.

In summary, we find that infant CTLs exert significantly stronger selection across *gag* and *nef* epitopes in early infection. In addition, we find that stronger selection across CTL epitopes is associated with favourable clinical outcomes. These results highlight the potential functionality of infant CTLs despite a lack of viral control with implications for the development of protective paediatric HIV-1 vaccines.

## Methods

### Cohort characteristics, sampling and ethics

Samples were collected, as part of the CTLs and the Prevention of HIV-1 Transmission Study, in Nairobi, Kenya between 1999 and 2002^41^. As described in detail elsewhere^41^, the parent cohort consisted of 465 HIV-1-infected women recruited during pregnancy and followed for up to two years postpartum with their infants. Mothers were provided with short-course zidovudine (ZDV) during the final trimester of pregnancy to reduce the risk of HIV-1 transmission according to contemporaneous guidelines^42^.In the absence of a national ART programme for children, all infants were treatment-naïve during the study period. Maternal and infant CD4 T cell counts and RNA viral loads were serially assessed at birth, and months 1, 3, 6, 12, 15, 18, 21, and 24. Infant viral loads were measured from cryopreserved plasma specimens, using the Gen-Probe assay^43^ (limit of detection of 50 copies/ml). Infant HIV-1 infection was defined as the first detection of HIV-1-*gag* DNA by PCR from dried blood spots^44^ or HIV-1 RNA from plasma^43^. Infection and classification into *in utero*, *peripartum*/early breast milk, or late breast milk transmission were defined as previously described^41^. Ninety transmission events were detected, with timing of infection available for 87 infants. Infant HLA types to two digit resolution were determined as previously described^45^. For evolutionary analyses, infants were selected if they were infected by one month of age and if they had three of more longitudinal plasma specimens available for study. Seventy two infants were infected by one month of age and 23 infants had samples from three or more longitudinal time points totalling 89 plasma specimens.

### Ethics statement

This study was conducted according to the principles expressed in the Declaration of Helsinki and the study protocol was approved by the Kenyatta National Hospital Ethics Review Committee and the Institutional Review Board of the University of Washington. HIV-1 seropositive pregnant women were enrolled after provision of written informed consent as detailed previously^41^.

### HIV-1 amplification and sequencing

As detailed in the Supplementary methods, viral RNA was isolated from cryopreserved plasma samples and purified using the QIAamp Viral RNA extraction kit (Qiagen, Limburg, Netherlands) followed by reverse transcription and nested PCR (Table S2) for *gag*, *pol* and *nef*. Amplified DNA was purified and sequenced on a 48 capillary ABI-3730 DNA analyser using Applied Biosystems BigDye Terminator v3.1 (Waltham, MA, USA) chemistry. Contigs were assembled and open reading frames edited using Geneious v6.1.6^46^ (Biomatters Ltd.). Full length *gag* and *nef* sequences corresponding to HXB2 nucleotide positions 790–2289 and 8797–9414, respectively, were obtained (except for *gag* sequences from infant 313, HXB2 positions 880–2289) as well as near full length *pol* sequences (2.0–2.8 kb spanning the protease through to integrase coding sequences, HXB2 positions 2358–5096). Ambiguous bases were identified using the Find Heterozygotes tool packaged in Geneious v6.1.6 and defined as peaks with quality scores >10 with a secondary peak height above 25% of the maximal peak. GenBank accession numbers for the sequences are indicated in Table S4.

### HIV-1 subtyping

The earliest infant sample following infection was used for subtyping and *gag*, *pol* and *nef*, sequences were analysed separately. Pairwise alignments of infant samples were made using the version and settings of MUSCLE^47^ packaged in Geneious v6.1.6. Profile alignments of infant sequences and the Los Alamos National Library HIV sequence database subtype reference alignment (2010)^48^ were made in Clustal X2^49^. All alignments were manually inspected and edited in MEGA v5.2^50^. Maximum-likelihood phylogenetic reconstruction (GTR + γ + I) with branch support estimated by the Approximate Likelihood Ratio Test (aLRT)-Shimodaira-Hasegawa-like (SH) procedure^51^ in PhyML 3.0 was carried out. Branches with aLRT-SH values above 0.85 were considered as statistically well supported. Sequences that either clustered with a known circulating recombinant form (CRF), clustered within different subtypes in distinct genetic regions, showed intermediate clustering across distinct genetic regions or presented long branches within a cluster were analysed as recombinant subtypes. Characterisation, using Simplot and Bootscan analysis in Simplot v3.5.1^52^, was carried out as detailed in the Supplementary methods.

### Phylogenetic estimation of intra-patient rates of evolution in *gag*, *pol* and *nef*

We estimated evolutionary rates separately for each individual infant using a maximum likelihood (ML) approach implemented in the HyPhy package^53^ with suitable modifications needed to analyse *gag, pol* and *nef*, respectively^17^,^54^. The Tamura-Nei (TN93) model of nucleotide evolution and a strict molecular clock were used to estimate evolutionary rates based on the procedure described by Hightower *et al*.^17^, having determined that rate estimates in all genes were unaffected by the complexity of the evolutionary model (Fig. S4). Briefly, sequences from each individual were aligned, and equipped with a “linear” tree, where the sequence at the oldest time point as the root, the second oldest sequence as the direct descendant of the root, and so on. Sequences from all individuals were analysed both individually (one individual at a time), and jointly (all individuals together) by profile likelihood in the ML phylogenetic framework. The likelihood ratio test (LRT) was used to test for deviations from molecular clock in a given individual, assuming the χ^2^_N−2_ asymptotic distribution of the test statistic (N = number of time points). Estimates of codon substitution rates, and the d*N*/d*S* ratio were obtained using the Muse-Gaut 94 x REV (MG94) codon-based model of evolution^55^. Infant HLA types at two-digit resolution and the 2013 LANLDB annotation of CTL-restricted epitopes^56^ were used to partition *gag, pol* and *nef* sequences into regions restricted by infant CTL and those that were not. The software pipeline implementing the analysis can be downloaded from https://github.com/spond/PolEvolution.

### Data analysis

Comparisons of global ML estimates of the evolutionary rates between HIV-1 genes were carried out using the Mann-Whitney U test. Two-sided p-values were calculated for analysis and values of p < 0.05 were considered significant. Correlations were tested using the Spearman’s rank correlation coefficient. Statistical analysis and plots were made in GraphPad Prism version 6.0 (GraphPad Software inc.). Severe immunosuppression was defined as a nadir CD4% ≤15 at any time point up to 15 months of age due to the association with infant mortality within 2 years of life in this cohort^57^. The Holm-Ŝidák test was used to correct for multiple comparisons.

## Additional Information

**How to cite this article**: Garcia-Knight, M. A. *et al*. Viral Evolution and Cytotoxic T Cell Restricted Selection in Acute Infant HIV-1 Infection. *Sci. Rep.* **6**, 29536; doi: 10.1038/srep29536 (2016).

## Supplementary Material

Supplementary Information

## Figures and Tables

**Table 1:** Infant characteristics.

Infant ID	Sex	MOT	Peak VL*	CD4% nadir† (TP)	Age last seen, mo	#Of sequences/TP			HLA A; B; C	Subtype
						*gag*	*pol*	*nef*		
135	M	IU	7.65	2 (4)	15	4	0	4	32, 6802; 39, 44; 04, 12	A1
159	F	P	6.94	18 (4)	24	6	1	4	29, 30; 4501, 4501; 06, 06	A1
168	M	P	8.15	6 (4)	18	6	3	6	24, 29 ;15, 5802; 02, 04	URF
170	M	IU	5.94	17 (3)	18	4	4	4	30, 3402; 4201/2, 57; 07, 17	A1
211	M	P	7.63	17 (4)	24	6	4	6	30, 6802; 4201/2, 4201/2; 07, 17	URF
231	F	P	6.26	18 (4)	24	4	4	4	26, 34; 35, 53; 04, 04	URF
258	M	IU	6.68	20 (3)	12	4	4	2	03, 30; 15, 7301;15, 17	A1
259	M	P	7.24	34 (4)	24	3	4	3	23, 74; 58, 4501; 07, 07	A1
261	F	P	6.57	16 (4)	24	5	3	5	2, 29; 5802, 35; 06, 07	URF
281	M	P	6.60	21 (3)	12	4	3	4	23, 74; 15, 15; 02, 02	A1
291	M	P	7.23	9 (6)	15	6	4	6	29, 74; 4201/2, 15; 02, 17	A1
303	M	P	7.62	17 (4)	12	2	3	1	30, 30; 4501, 4501; 1601, 1601	D
313	M	IU	7.45	12 (6)	24	4	2	3	33, 6802; 15, 35; 03, 04	URF
334	F	P	6.46	24 (4)	9	4	0	4	29, 26/66; 13, 15; 02, 06	A1
411	F	P	7.15	15 (5)	24	4	4	4	30, 30; 15, 42; 14, 17	A1
424	F	IU	7.45	7 (2)	6	3	2	3	02, 6802; 51, 08; 07, 1601	URF
440	M	IU	6.88	10 (3)	10	4	1	3	02, 30; 4501/03, 4902; 06, 1601	A1
454	M	P	7.64	23 (5)	24	4	4	4	02, 31; 08, 15; 07, 08	URF
485	M	IU	6.39	4 (7)	24	4	3	2	NA; NA; NA	A1

^*^Maximum VL measurement within 6 months of infection, log_10_ HIV-1 plasma RNA copies/mL of plasma.

^†^Within 15 months of age. TP, time points; NA, not available; VL, viral load; mo, months; MOT, mode of transmission; IU, *in utero*; P, *peripartum*; URF, unique recombinant form, parental subtypes shown in S3 Table.

**Table 2:** Global evolutionary rates and dN/dS rate ratios in infants stratified by clinical parameters.

Gene	Immunosuppression			Peak VL			Mode of transmission			HIV subtype		
	NSIS	SIS	*P* (LRT)	< Median	> Median	*P* (LRT)	*in utero*	*peripartum*	*P* (LRT)	A1	Other	*P* (LRT)
	N = 10	N = 8		N = 9	N = 9		N = 7	N = 11		N = 11	N = 7	
*gag*†rate, (95% CI)	4.2 (3.0–5.0)	8.8 (7.0–11.0)	**0.008** (**19.6**)	6.1 (5.0–7.0)	5.8 (5.0–7.0)	0.8 (0.07)	6.7 (5.0–9.0)	5.7 (5.0–7.0)	0.4 (0.8)	6.0 (5.0–7.0)	5.9 (5.0–8.0)	1.0 (0.0)
*gag* d*N*/d*S*, (95% CI)	1.4 (1.1–1.8)	0.4 (0.3–0.5)	**0.006** (**10.4**)	0.9 (0.7–1.2)	0.5 (0.4–0.7)	0.08 (3.0)	0.4 (0.3–0.6)	0.8 (0.6–1.0)	0.1 (2.4)	0.7 (0.5–0.8)	0.6 (0.5–0.9)	0.8 (0.05)
	N = 9	N = 4		N = 9	N = 4		N = 3	N = 10		N = 7	N = 6	
*pol* rate (95% CI)	1.4 (1.0–2.0)	2.1 (1.0–3.0)	0.2 (1.8)	1.4 (1.0–2.0)	1.6 (1.0–2.0)	0.7 (0.2)	1.0 (0.0–2.0)	1.6 (1.0–2.0)	0.3 (0.9)	1.5 (1.0–2.0)	1.6 (1.0–2.0)	0.9 (0.02)
*pol* d*N*/d*S* (95% CI)	0.3 (0.2–0.5)	0.3 (0.1–0.5)	1.0 (0.00)	0.2 (0.1–0.4)	0.4 (0.2–0.6)	0.3 (1.2)	1.1 (0.2–2.8)	0.2 (0.1–0.4)	0.2 (1.9)	0.4 (0.2–0.7)	0.2 (0.09–0.4)	0.2 (1.4)
	N = 9	N = 7		N = 7	N = 9		N = 5	N = 11		N = 9	N = 7	
*nef* rate, (95% CI)	8.0 (6.0–11)	19.4 (15.0–25.0)	**0.008** (**20.2**)	6.7 (4.0–10.0)	16.6 (13.0–20.0)	**0.007** (**17.3**)	14.1 (9.0–20.0)	12.1 (10.0–15.0)	0.5 (0.5)	9.0 (6.0–12.0)	16.1 (13.0–20.0)	**0.018** (**8.74**)
*nef* d*N*/d*S*, (95% CI)	1.1 (0.8–1.5)	0.6 (0.4–0.8)	0.2 (1.8)	0.7 (0.4– 1.2)	0.8 (0.6–1.0)	1.0 (0.00)	0.4 (2.0–0.6)	1.0 (0.8–1.3)	0.03(4.9)	0.8 (0.5–1.1)	0.8 (0.6–1.0)	1.0 (0.0)

*Median of N = 19 infants. ^†^Rate = nucleotide substitutions/1000 sites/year. NSIS, non-severely immunosuppressed (CD4% >15 during follow-up). SIS, severely immunosuppressed (CD4% ≤15 during follow-up). VL, viral load; LRT, likelihood ratio test. Bold indicates significant values after correction for multiple comparisons.

**Table 3:** d*N*/d*S* rate ratios across *gag* and *nef* in HLA and non-HLA targeted epitopes in infants stratified by immunosuppression.

Gene	Parameter	d*N*/d*S* ratio (95% CI)			
		CTL+	CTL-	*P*	LRT
*gag*	NSIS (N = 10)	2.3 (1.6–3.3)	1.0 (0.7–1.5)	**0**.**006**	7.6
	SIS (*N = 7)	0.5 (0.3–0.8)	0.4 (0.3–0.5)	0.717	0.1
*nef*	NSIS (N = 9)	2.6 (1.7–3.8)	0.5 (0.2–0.8)	**<0**.**001**	20.4
	SIS (N = 7)	0.7 (0.4–1.1)	0.6 (0.4–0.8)	0.525	0.4

SIS, severely immunosuppressed; NSIS, non-severely immunosuppressed; LRT, likelihood ratio test. *HLA data missing from infant 485.
