# Supplementary material for: Viral Evolution and Cytotoxic T Cell Restricted Selection in Acute Infant HIV-1 Infection
Source: Sci Rep. 2016 Jul 12;6:29536. doi: 10.1038/srep29536 (PMC4941567; doi:10.1038/srep29536)
Supplement: Supplementary Information [file srep29536-s1.pdf]

Supplementary information

Viral Evolution and Cytotoxic T Cell Restricted Selection in Acute Infant HIV-1 Infection

Miguel A. Garcia-Knight, Jennifer Slyker, Barbara Lohman Payne, Sergei L Kosakovsky Pond, Thushan I. de Silva, Bhavna Chohan, Brian Khasimwa, Dorothy Mbori-Ngacha, Grace John-Stewart, Sarah L. Rowland-Jones, Joakim Esbjörnsson.

S1 Text. Materials and Methods

Viral RNA extraction, reverse transcription, PCR amplification and purification of full length *gag, pol* and *nef*. The QIAamp Viral RNA extraction kit (Qiagen, Limburg, Netherlands) was used to purify viral RNA from 140µl of peripheral blood plasma following the manufacturer's instructions. Purified RNA was stored at -80˚C until use. HIV-1 *gag, pol* and *nef* sequences were amplified by nested PCR(1). The Titan One tube RT-PCR Kit (Roche, Basel, Switzerland) was used for reverse transcription (RT) and first round PCR amplification of *gag, pol* and *nef* in a single reaction. The Expand High Fidelity PCR System (Roche) was used to amplify the 1st round PCR products. All primers for PCR and sequencing (S2 Table) were synthesised by MWG Operon (Huntsville, AL, USA). For RT of *gag* and 1st round amplification, in each 25µL reaction, 1x reaction buffer, 200µM dNTPs, 0.5mM of each primer CTLGagOF and CTLGag OR (Table S2), 5U of RNAse inhibitor, 0.5µL of enzyme mix and 5µL of extracted viral RNA were used. RT was carried out at 50˚C for 30 min, followed by 94˚C for 2 min, followed by 10 cycles of 94˚C for 15 sec, 54˚C for 30 sec and 68˚C for 2 min, followed by 19 cycles of 94˚C for 15 sec, 54˚C for 30 sec and 68˚C for 2 minutes with 5 sec increments at each cycle and a final extension at 68˚C for 7 min. For 2nd round *gag* amplification, nested primers CTLGagIF and CTLGagIR (Table 2) at 0.3µM were used in a 50µL reaction together with 1X reaction buffer, 200µM dNTPs, 0.75µL of enzyme mix and 1uL of first round product as template. The reaction proceeded under the same conditions as the 1st round reaction omitting the RT step. For *pol*, the 1st and 2nd round reactions were set up with primers pairs CTLPolOF and CTLPolOR, and CTLPolIF and CTLPolIR (S2 Table) respectively at 0.2µM each in both reactions. All other reaction components were the same as detailed for *gag* for both reactions. 1st and 2nd round reactions proceeded under the same cycling conditions as for *gag* except the first two elongation steps proceeded for 3 min. For *nef*, the 1st and 2nd round reactions were set up

with primers pairs CTLNefOF and CTLNefOR, and CTLNefIF and CTLNefIR (Table S2) respectively at 0.2µM each in both reactions. In the 1st round reaction RT proceeded at 50˚C for 30 min and 94˚C for 2 min then 40 cycles of 94˚C for 15 sec, 54˚C for 30 sec and 72˚C for 2 min with a final extension at 72˚C for 7 min. The second round reaction proceeded under the same cycling conditions as the 1st round omitting the RT step. Nested product were resolved on a 1% agarose gel. Amplicons yielding a single sized band were directly purified using the PCR purification Kit (Qiagen). Amplicons resulting in multiple sized bands were gel purified using the QIAquick Gel Extraction Kit (Qiagen). Purified DNA was stored at -20˚C.

Recombinant subtype characterisation. A concatenated alignment (5009 bp) of near full length *gag-pol-nef* infant sequences and the full HIV-1 LANLDB subtype reference alignment was made and gap stripped. A sliding window of 400bp was used with increments of 50bp. Bootscan analysis was carried out using the neighbour-joining method with the Kimura 2-parameter model, a transition/transversion rate of 2.0 and with 100 bootstrap replicates for each sliding window. Breakpoint coordinates relative to HXB2 were identified using the HIV Sequence Locator Tool(2) at the LANLDB. The Recombinant HIV-1 Drawing Tool(2) from the LANLDB was used to depict the breakpoint positions on the HIV-1 genome.

Supplementary Tables and Figures

S1 Table. Comparison of baseline characteristics in infants included in the evolutionary analysis and those with early HIV infection in the parent cohort

Number of infants	19	72	*P*
Male (%)	13 (68)	36 (50)	0.2†
In utero transmission (%)	7 (37)	28 (39)	1.0†
*Peripartum/*early breast feeding transmission (%)	12 (63)	44 (61)	1.0†
Median peak $\log_{10}$ HIV-1 RNA copies/mL plasma (IQR)	7.2 (6.6-7.6)	6.8 (6.4-7.4)	0.3‡
Median CD4 count in cells/mL (IQR) at 6 months	1415 (760-2195)	*1236 (780-2052)	0.7§
Median CD4% (IQR) at 6 months	22.0 (16.0-26.0)	*20.5 (11.5-28.0)	0.9‡
A1 (%)	11 (58)	NA	-
D (%)	1 (5)	NA	-
URFs (%)	7 (37)	NA	-

*Data available for 42 infants with early infection. †Fisher's exact test. ‡Unpaired t test. §Mann-Whitney U test. NA, not available.

S2 Table. Primers used to amplify and sequence HIV-1 *gag*, *pol* and *nef*

Product	Primer	Sequence (5'-3')	Location relative to HXB2
gag	CTLGagOF	GTTCTCTCGACGCAGGACTC	680-699
	*CTLGagIF	AGCGGAGGCTAGAAGGAGAG	768-787
	CTLG01	ATCGTTCTAGCTCCCTGCTT	919-900
	CTLG00	GCATGGGTAAAAGTAGTAGAAGA	1249-1271
	CTLG03	ACTCTATCCCATTCTGCAGC	1433-1414
	CTLG02	TAGAAGAAATGATGACAGCATG	1817-1838
	CTLG05	TATGTGCCCTTCTTTGCCAC	1991-1973
	CTLG110	AGGCTAATTTTTTAGGGA	2078-2095
	*CTLGagIR	AACCTCCAATTCCCCCTATC	2409-2390
	CTLGagOR	CCAATTATGTTGACAGGTGTAGG	2509-2487
pol	CTLPolOF	TCCCTCAAATCACTCTTTGG	2251-2270
	*CTLPolIF	GCTCTATTAGATACAGGAGCAGATG	2316-2340
	CTLGagOR	CCAATTATGTTGACAGGTGTAGG	2509-2487
	CTLP00	GCCTGAAAATCCATACAATACTCC	2702-2725
	CTLP01	AATATGCATCACCCACATC	2895-2877
	CTLP02	CAGTACAGCCTATAGTGCTGCCA	3268-3290
	CTLP03	GCCAATTCTAATTCTGCTTC	3460-3441
	CTLP04	AGTGGGAGTTTGTCAATACC	3787-3806
	CTLP05	ACTACAGTCTACTTGTCCATG	4400-4380
	CTLP06	CACAAAGGAATTGGAGGAAATG	4164-4185
	CTLP07	GAGCTTTGCTGGTCCTTTCC	4952-4933
	CTLP08	TAAGACAGCAGTACAAATGGCAG	4745-4767
	*CTLPolIR	TAGTGGGATGTGTACTTCTGAACT	5217-5194
	AJB-1R	TATGGATTTTCAGGYCCAATTYTTG	2725-2702
	AJB-4F	ACACCAGAYAARAARCATCAGAAAG	3195-3219
	AJB-3R	TTCTGTATRTCATTGACAGTCCAGCT	3325-3300
	AJB-5R	GATTCCTAATGCATACTGTGAGTCTG	4064-4039
	CTLPolOR	TGTATGCAGACCCCAATATGT	5262-5242
nef	CTLNefOF	TGTGCCTCTTCAGCTACCAC	8512-8531
	*CTLNefIF	CGAGGACTGTGGAACTTCTGG	8560-8680
	CTLNef00	ACACAAGGCTACTTCCCTGA	9557-9533
	CTLNef01	GTGTAATTCTGCCAATCAGGGA	9145-9164
	*CTLNefIR	GGTCTAACAAGAGAGACCCAGTACA	9179-9158
	CTLNefOR	CCCAGGCTCGATCTGGTC	9572-9554

*Inner primers from the nested PCR reaction were used to sequence the 3' and 5' amplicon ends.

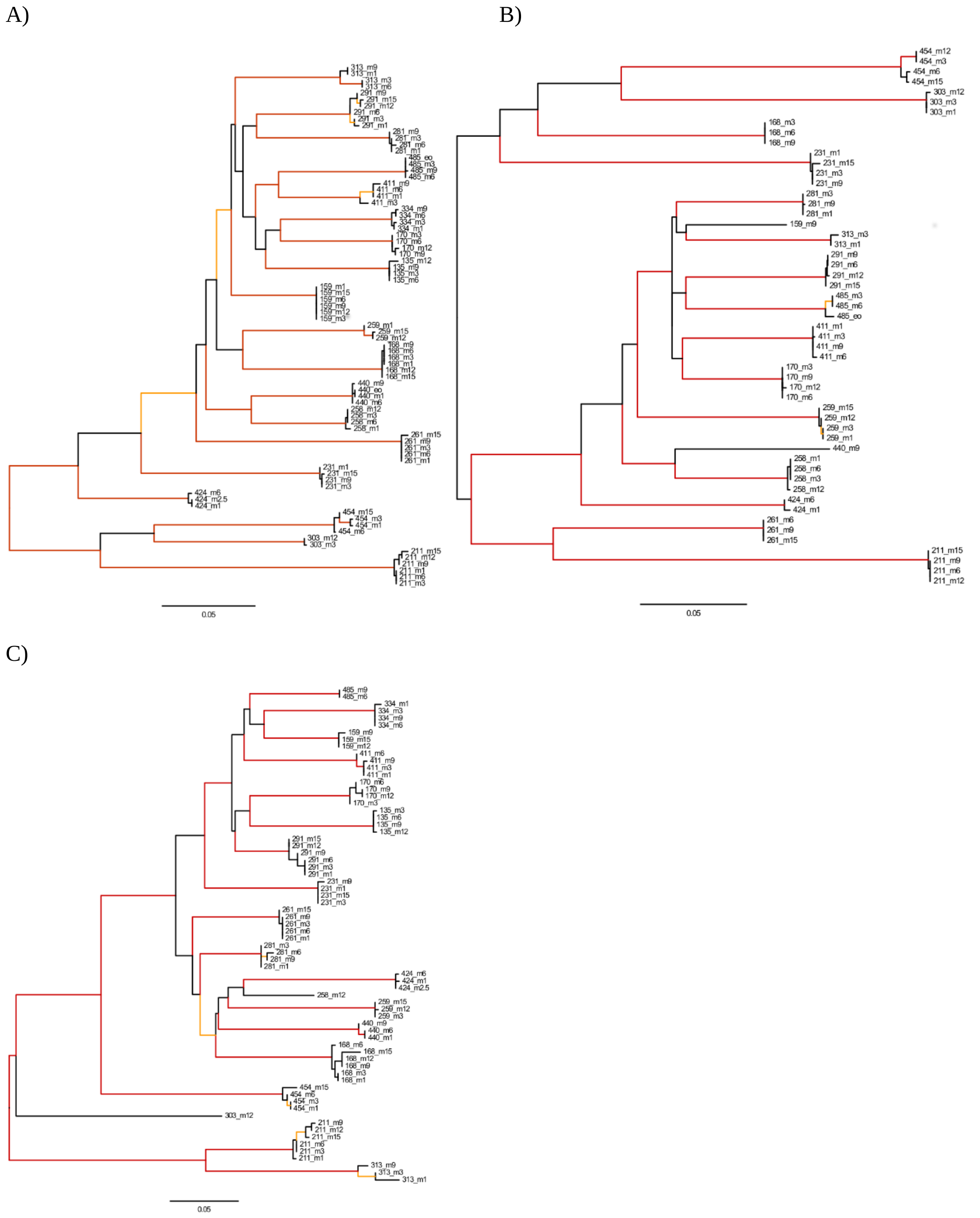

S1 Fig. Patient specific cluster analysis of infant HIV-1 sequences sampled longitudinally. Maximum likelihood trees representing *gag* (A), *pol* (B) and *nef* (C) sequences. The General Time Reversible model of nucleotide substitutions with proportion of invariable sites and substitution rate heterogeneity was used. Branch support was estimated using the aLRT-SH procedure; branches with support values >0.85 are shown in yellow and >0.9, in red. Tip labels indicate infant ID number and the month (m) of the sample.

A)

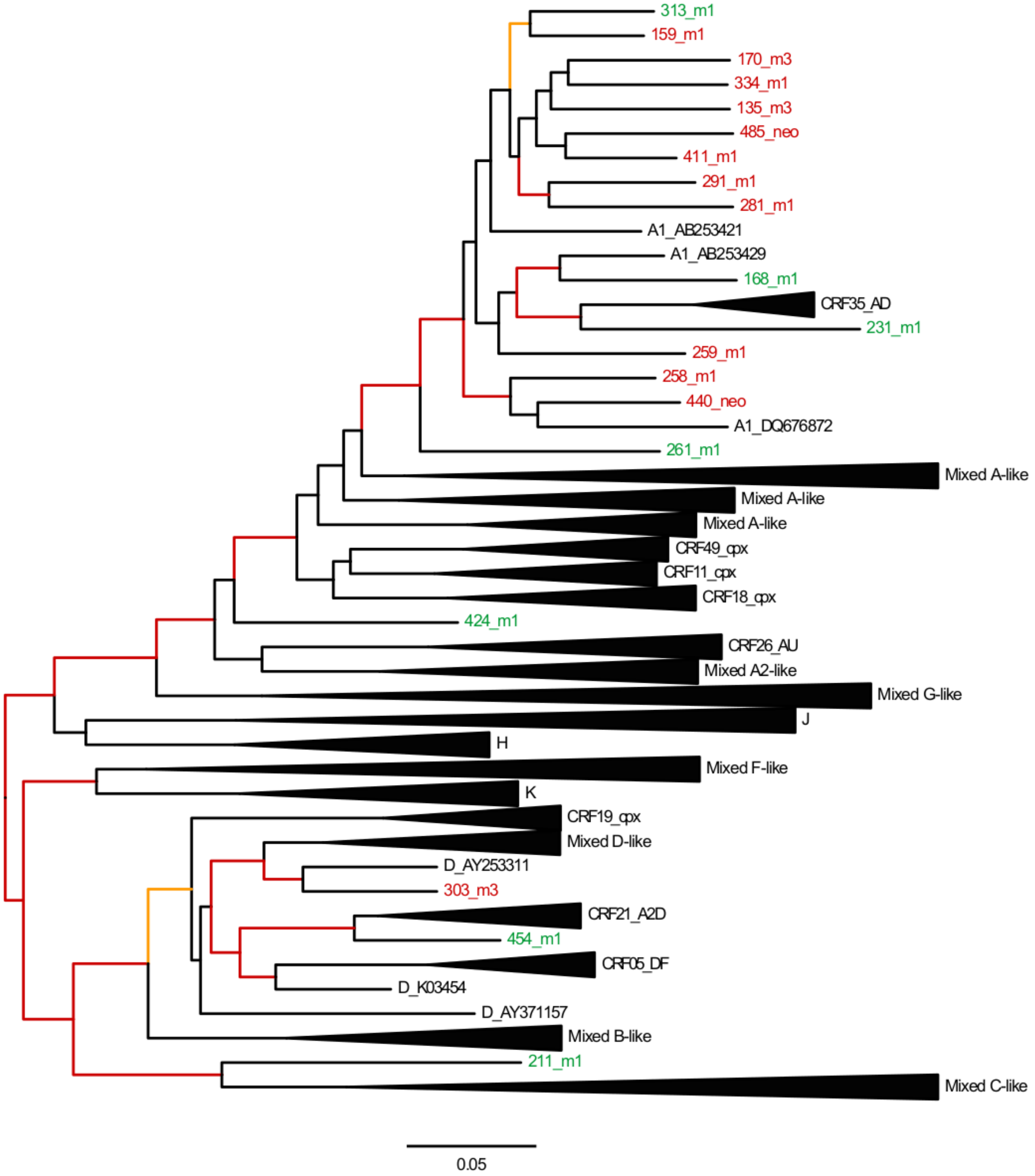

313_m1
159_m1
170_m3
334_m1
135_m3
485_neo
411_m1
291_m1
281_m1
A1_AB253421
A1_AB253429
168_m1
CRF35_AD
231_m1
259_m1
258_m1
440_neo
A1_DQ676872
261_m1
Mixed A-like
Mixed A-like
Mixed A-like
CRF49_cpx
CRF11_cpx
CRF18_cpx
424_m1
CRF26_AU
Mixed A2-like
Mixed G-like
J
H
Mixed F-like
K
CRF19_cpx
Mixed D-like
D_AY253311
303_m3
CRF21_A2D
454_m1
CRF05_DF
D_K03454
D_AY371157
Mixed B-like
211_m1
Mixed C-like
0.05

B)

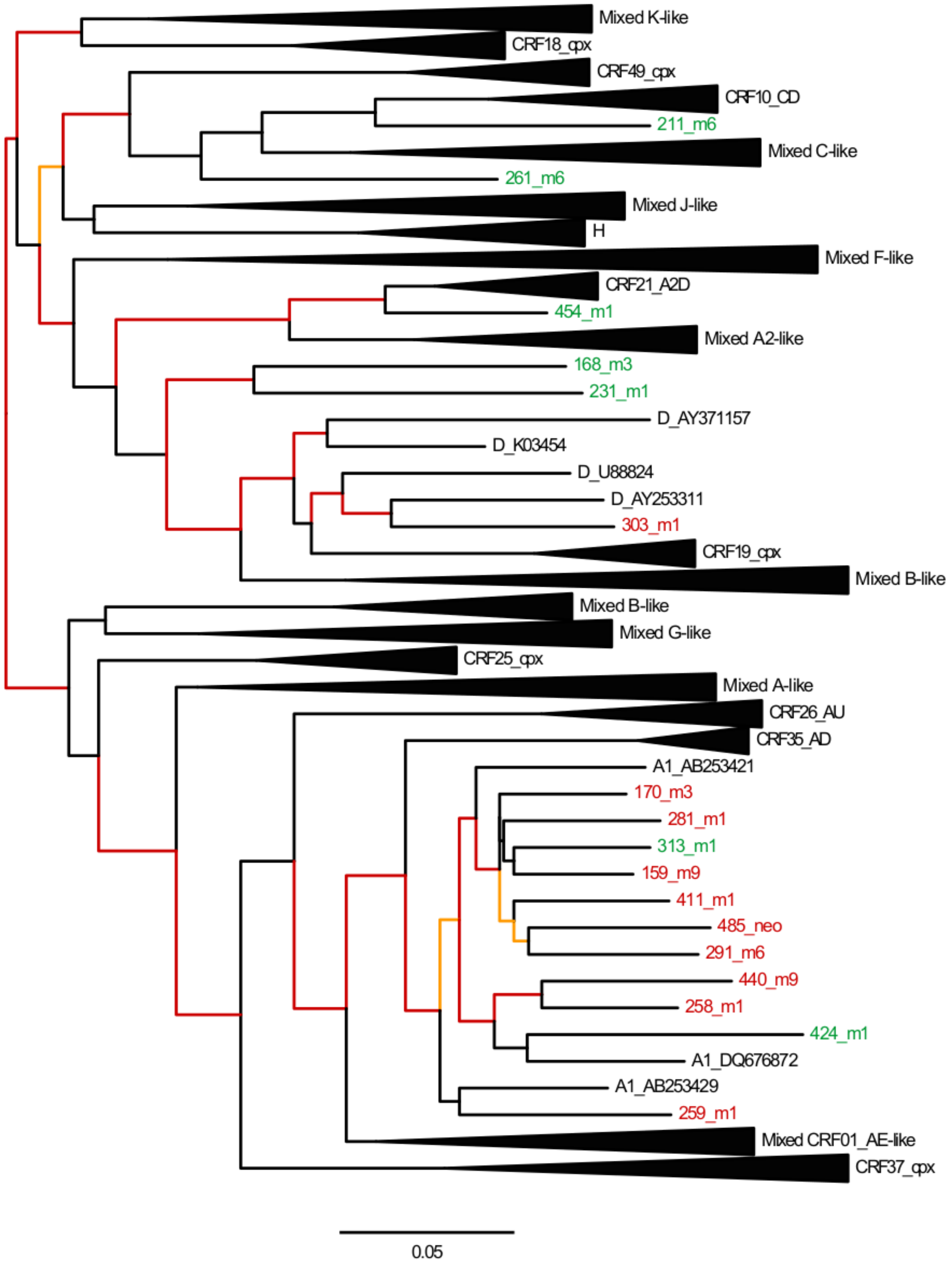
Mixed K-like
CRF18_cpx
CRF49_cpx
CRF10_CD
211_m6
Mixed C-like
261_m6
Mixed J-like
H
Mixed F-like
CRF21_A2D
454_m1
Mixed A2-like
168_m3
231_m1
D_AY371157
D_K03454
D_U88824
D_AY253311
303_m1
CRF19_cpx
Mixed B-like
Mixed B-like
Mixed G-like
CRF25_cpx
Mixed A-like
CRF26_AU
CRF35_AD
A1_AB253421
170_m3
281_m1
313_m1
159_m9
411_m1
485_neo
291_m6
440_m9
258_m1
424_m1
A1_DQ676872
A1_AB253429
259_m1
Mixed CRF01_AE-like
CRF37_cpx
0.05

C)

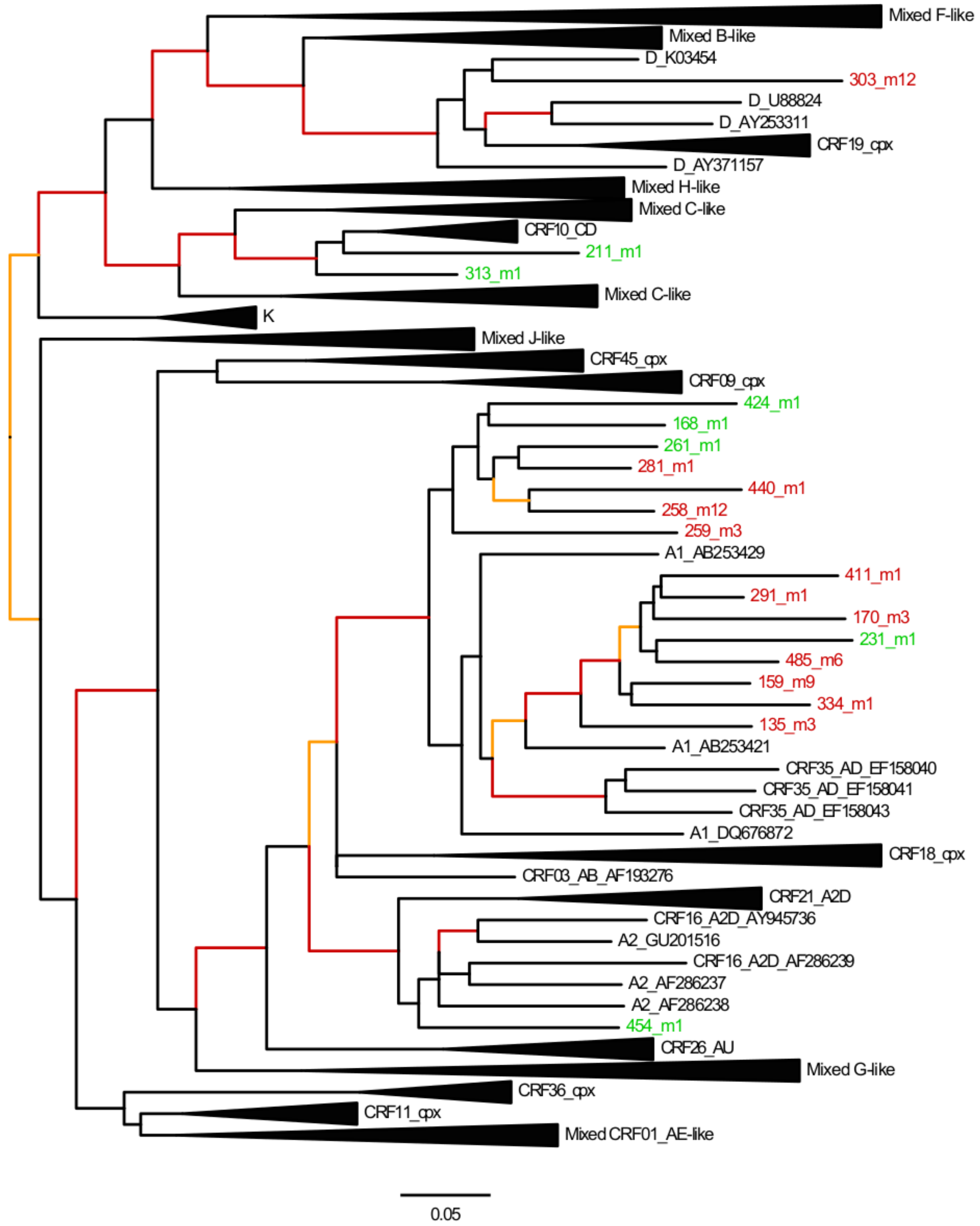

S2 Fig. Phylogenetic subtype classification of infant-derived *gag, pol* and *nef* HIV-1 sequences. Maximum likelihood phylogenetic trees built from *gag* (A; n=19) *pol* (B; n=17) and *nef* (C; n=19) infant sequences (infant ID and month of age shown) and the full Los Alamos (2010) subtype reference dataset. The General Time Reversible (GTR) model of nucleotide substitution, with proportion of invariable sites and substitution rate heterogeneity was used.

Branch support was estimated by the Approximate Likelihood Ratio Test (aLRT)-Shimodaira-Hasegawa-like (SH) procedure; branches with support values >0.85 are shown in yellow and >0.9 in red. All collapsed clusters were supported by aLTR-SH >0.85. Sequences from the earliest infant sampling time point were used. HIV-1 strains that showed corresponding results throughout the three analysed genetic regions are shown in red and HIV-1 strains with conflicting results between genetic regions are shown in green.

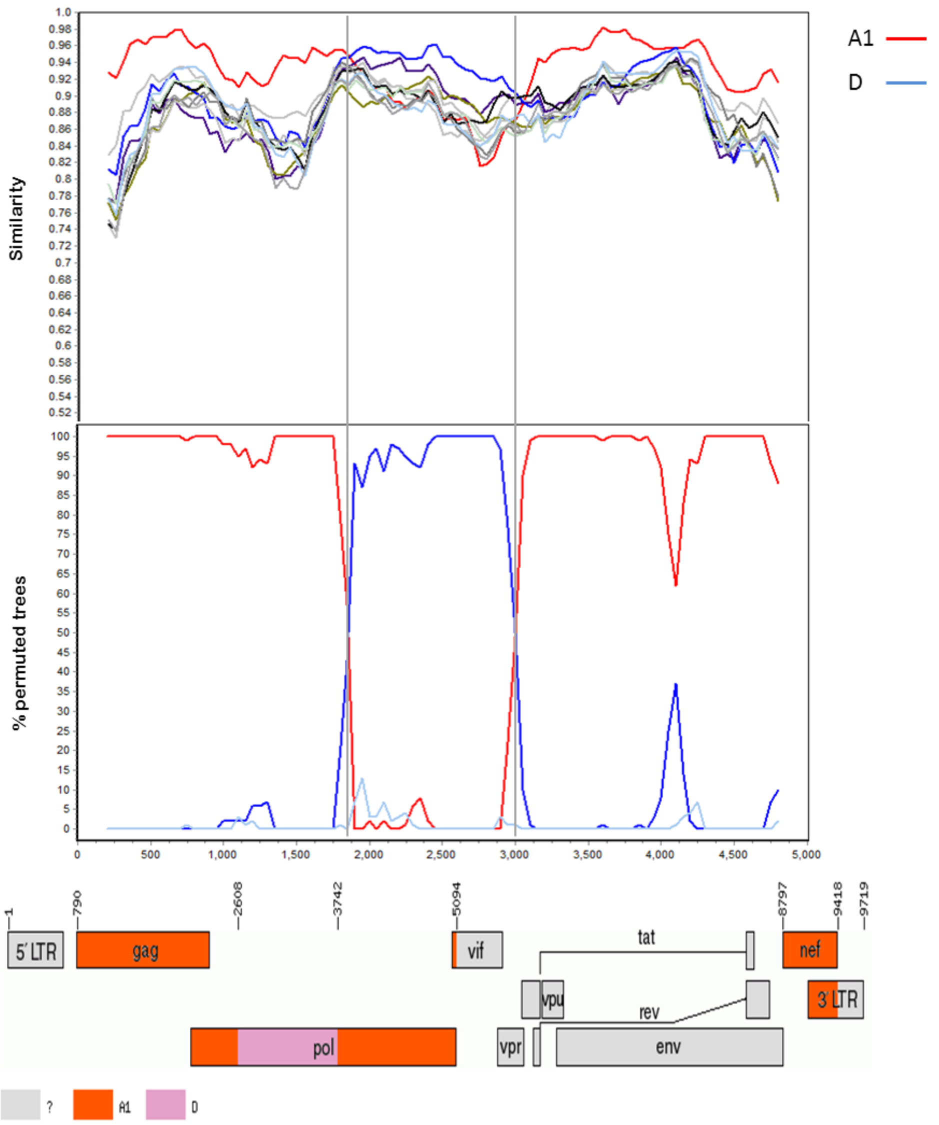

S3 Fig. Recombination analysis of the HIV-1 viral sequence from infant 168. Simplot (upper plot) and Bootscan (lower plot) analysis was carried on concatenated *gag*, *pol* and *nef* sequences of the viral isolate from infant 168 to assess subtype recombination. Recombination breakpoints are indicated with vertical grey lines and the positions were mapped onto the HIV-1 genome using the LANL database Recombinant HIV-1 Drawing Tool (below). The figure depicts a double recombination event within *pol* produced an A1D recombinant; the A1 subtype corresponded to the 3’ region of *pol* and this was also found for *nef*.

S3 Table. Characteristics of recombinant HIV-1 viral sequences following subtyping analysis

Patient	**Parental subtypes**	**Breakpoints relative to start of HXB2 (bp)**
168	A1, D	*1383*, 2720, 4362
211	CRF10, C	*1325*, 2920
231	A1, D	*1865*, 3494, 4124. 4814
261	A1, C	3361, 3776, 3869, 4887
313	A1, CRF10	4931
424	A1, A2, D	*1284*, *2173*, 2643, 4264, 4767
454	CRF21, A2	4984

Breakpoint within *gag* shown in italics, otherwise breakpoint in *pol*

A)

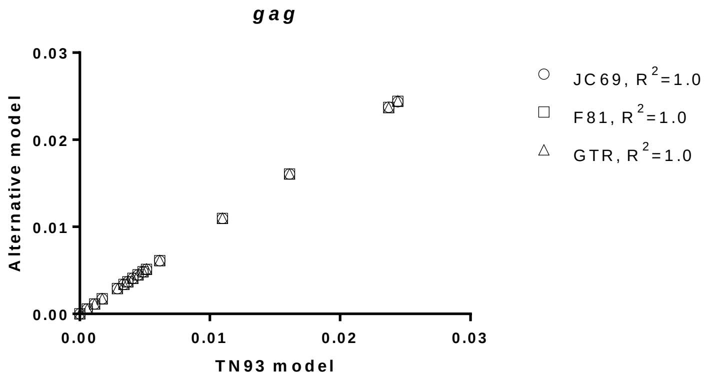

B)

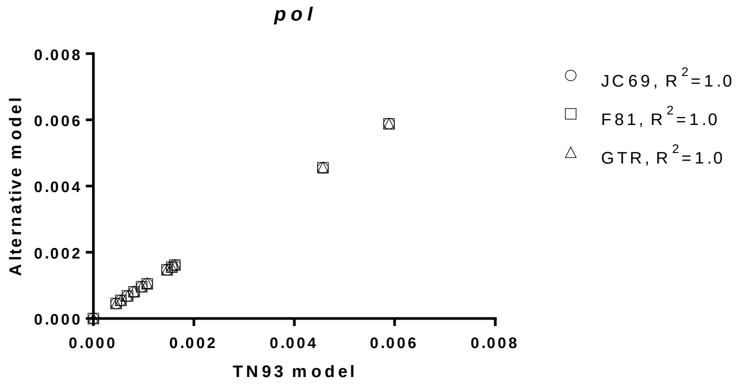

C)

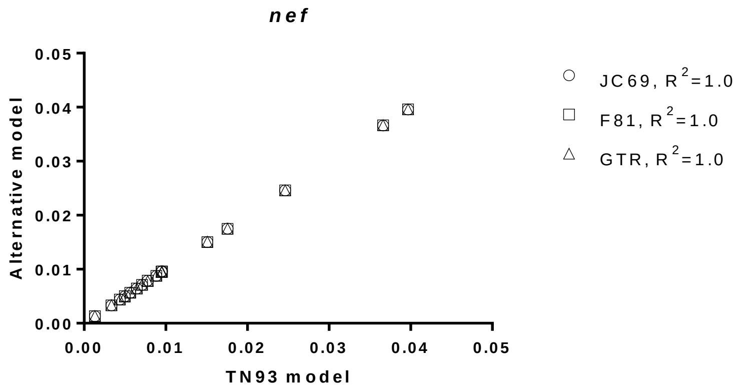

S4 Fig. Substitution rate estimates are unaltered when different evolutionary models are applied. A perfect linear correlation was observed between individual rate estimates for *gag* (A), *pol* (B) and *nef* (C) under the TN93 model of nucleotide substitution and three alternative models with varying complexity, from the simplest (JC69) to the most general (GTR).

S4 Table. Sequences used in evolutionary analyses

Accession number	Sequence name	Infant ID	Sample time point	HIV-1 gene	Sample year
KX302403	00KE135gag.M3	135	M3	gag	2000
KX302404	00KE135gag.M6	135	M6	gag	2000
KX302405	00KE135nef.M3	135	M3	nef	2000
KX302406	00KE135nef.M6	135	M6	nef	2000
KX302407	00KE159gag.M1	159	M1	gag	2000
KX302408	00KE159gag.M3	159	M3	gag	2000
KX302409	00KE168gag.M1	168	M1	gag	2000
KX302410	00KE168gag.M3	168	M3	gag	2000
KX302411	00KE168nef.M1	168	M1	nef	2000
KX302412	00KE168nef.M3	168	M3	nef	2000
KX302413	00KE168pol.M3	168	M3	pol	2000
KX302414	00KE170gag.M3	170	M3	gag	2000
KX302415	00KE170gag.M6	170	M6	gag	2000
KX302416	00KE170nef.M3	170	M3	nef	2000
KX302417	00KE170nef.M6	170	M6	nef	2000
KX302418	00KE170pol.M3	170	M3	pol	2000
KX302419	00KE170pol.M6	170	M6	pol	2000
KX302420	00KE211gag.M1	211	M1	gag	2000
KX302421	00KE211gag.M3	211	M3	gag	2000
KX302422	00KE211nef.M1	211	M1	nef	2000
KX302423	00KE211nef.M3	211	M3	nef	2000
KX302424	00KE231gag.M9	231	M9	gag	2000
KX302425	00KE231nef.M9	231	M9	nef	2000
KX302426	00KE231pol.M9	231	M9	pol	2000
KX302427	01KE135gag.M12	135	M12	gag	2001
KX302428	01KE135gag.M9	135	M9	gag	2001
KX302429	01KE135nef.M12	135	M12	nef	2001
KX302430	01KE135nef.M9	135	M9	nef	2001
KX302431	01KE159gag.M12	159	M12	gag	2001
KX302432	01KE159gag.M15	159	M15	gag	2001
KX302433	01KE159gag.M6	159	M6	gag	2001
KX302434	01KE159gag.M9	159	M9	gag	2001
KX302435	01KE159nef.M12	159	M12	nef	2001
KX302436	01KE159nef.M15	159	M15	nef	2001
KX302437	01KE159nef.M9	159	M9	nef	2001
KX302438	01KE168gag.M12	168	M12	gag	2001
KX302439	01KE168gag.M15	168	M15	gag	2001
KX302440	01KE168gag.M6	168	M6	gag	2001
KX302441	01KE168gag.M9	168	M9	gag	2001
KX302442	01KE168nef.M12	168	M12	nef	2001
KX302443	01KE168nef.M15	168	M15	nef	2001
KX302444	01KE168nef.M6	168	M6	nef	2001

KX302445	01KE168nef.M9	168	M9	nef	2001
KX302446	01KE168pol.M6	168	M6	pol	2001
KX302447	01KE168pol.M9	168	M9	pol	2001
KX302448	01KE170gag.M12	170	M12	gag	2001
KX302449	01KE170gag.M9	170	M9	gag	2001
KX302450	01KE170nef.M12	170	M12	nef	2001
KX302451	01KE170nef.M9	170	M9	nef	2001
KX302452	01KE170pol.M12	170	M12	pol	2001
KX302453	01KE170pol.M9	170	M9	pol	2001
KX302454	01KE211gag.M12	211	M12	gag	2001
KX302455	01KE211gag.M15	211	M15	gag	2001
KX302456	01KE211gag.M6	211	M6	gag	2001
KX302457	01KE211gag.M9	211	M9	gag	2001
KX302458	01KE211nef.M12	211	M12	nef	2001
KX302459	01KE211nef.M15	211	M15	nef	2001
KX302460	01KE211nef.M6	211	M6	nef	2001
KX302461	01KE211nef.M9	211	M9	nef	2001
KX302462	01KE211pol.M12	211	M12	pol	2001
KX302463	01KE211pol.M15	211	M15	pol	2001
KX302464	01KE211pol.M6	211	M6	pol	2001
KX302465	01KE211pol.M9	211	M9	pol	2001
KX302466	01KE231gag.M1	231	M1	gag	2001
KX302467	01KE231gag.M3	231	M3	gag	2001
KX302468	01KE231nef.M1	231	M1	nef	2001
KX302469	01KE231nef.M3	231	M3	nef	2001
KX302470	01KE231pol.M1	231	M1	pol	2001
KX302471	01KE231pol.M3	231	M3	pol	2001
KX302472	01KE258gag.M1	258	M1	gag	2001
KX302473	01KE258gag.M3	258	M3	gag	2001
KX302474	01KE258gag.M6	258	M6	gag	2001
KX302475	01KE258pol.M1	258	M1	pol	2001
KX302476	01KE258pol.M3	258	M3	pol	2001
KX302477	01KE258pol.M6	258	M6	pol	2001
KX302478	01KE259gag.M1	259	M1	gag	2001
KX302479	01KE259nef.M3	259	M3	nef	2001
KX302480	01KE259pol.M1	259	M1	pol	2001
KX302481	01KE259pol.M3	259	M3	pol	2001
KX302482	01KE261gag.M1	261	M1	gag	2001
KX302483	01KE261gag.M3	261	M3	gag	2001
KX302484	01KE261gag.M6	261	M6	gag	2001
KX302485	01KE261nef.M1	261	M1	nef	2001
KX302486	01KE261nef.M3	261	M3	nef	2001
KX302487	01KE261nef.M6	261	M6	nef	2001
KX302488	01KE261pol.M6	261	M6	pol	2001
KX302489	01KE281gag.M1	281	M1	gag	2001
KX302490	01KE281gag.M3	281	M3	gag	2001

KX302491	01KE281gag.M6	281	M6	gag	2001
KX302492	01KE281nef.M1	281	M1	nef	2001
KX302493	01KE281nef.M3	281	M3	nef	2001
KX302494	01KE281nef.M6	281	M6	nef	2001
KX302495	01KE281pol.M1	281	M1	pol	2001
KX302496	01KE281pol.M3	281	M3	pol	2001
KX302497	01KE291gag.M1	291	M1	gag	2001
KX302498	01KE291gag.M3	291	M3	gag	2001
KX302499	01KE291nef.M1	291	M1	nef	2001
KX302500	01KE291nef.M3	291	M3	nef	2001
KX302501	01KE303pol.M1	303	M1	pol	2001
KX302502	01KE303pol.M3	303	M3	pol	2001
KX302503	01KE313gag.M1	313	M1	gag	2001
KX302504	01KE313gag.M3	313	M3	gag	2001
KX302505	01KE313nef.M1	313	M1	nef	2001
KX302506	01KE313nef.M3	313	M3	nef	2001
KX302507	01KE334gag.M1	334	M1	gag	2001
KX302508	01KE334nef.M1	334	M1	nef	2001
KX302509	02KE231gag.M15	231	M15	gag	2002
KX302510	02KE231nef.M15	231	M15	nef	2002
KX302511	02KE231pol.M15	231	M15	pol	2002
KX302512	02KE258gag.M12	258	M12	gag	2002
KX302513	02KE258pol.M12	258	M12	pol	2002
KX302514	02KE259gag.M12	259	M12	gag	2002
KX302515	02KE259gag.M15	259	M15	gag	2002
KX302516	02KE259nef.M12	259	M12	nef	2002
KX302517	02KE259nef.M15	259	M15	nef	2002
KX302518	02KE259pol.M12	259	M12	pol	2002
KX302519	02KE259pol.M15	259	M15	pol	2002
KX302520	02KE261gag.M15	261	M15	gag	2002
KX302521	02KE261gag.M9	261	M9	gag	2002
KX302522	02KE261nef.M15	261	M15	nef	2002
KX302523	02KE261nef.M9	261	M9	nef	2002
KX302524	02KE261pol.M15	261	M15	pol	2002
KX302525	02KE261pol.M9	261	M9	pol	2002
KX302526	02KE281gag.M9	281	M9	gag	2002
KX302527	02KE281nef.M9	281	M9	nef	2002
KX302528	02KE281pol.M9	281	M9	pol	2002
KX302529	02KE291gag.M12	291	M12	gag	2002
KX302530	02KE291gag.M15	291	M15	gag	2002
KX302531	02KE291gag.M6	291	M6	gag	2002
KX302532	02KE291gag.M9	291	M9	gag	2002
KX302533	02KE291nef.M12	291	M12	nef	2002
KX302534	02KE291nef.M15	291	M15	nef	2002
KX302535	02KE291nef.M6	291	M6	nef	2002
KX302536	02KE291nef.M9	291	M9	nef	2002

KX302537	02KE291pol.M12	291	M12	pol	2002
KX302538	02KE291pol.M15	291	M15	pol	2002
KX302539	02KE291pol.M6	291	M6	pol	2002
KX302540	02KE291pol.M9	291	M9	pol	2002
KX302541	02KE303pol.M12	303	M12	pol	2002
KX302542	02KE313gag.M6	313	M6	gag	2002
KX302543	02KE313gag.M9	313	M9	gag	2002
KX302544	02KE313nef.M9	313	M9	nef	2002
KX302545	02KE334gag.M3	334	M3	gag	2002
KX302546	02KE334gag.M6	334	M6	gag	2002
KX302547	02KE334gag.M9	334	M9	gag	2002
KX302548	02KE334nef.M3	334	M3	nef	2002
KX302549	02KE334nef.M6	334	M6	nef	2002
KX302550	02KE334nef.M9	334	M9	nef	2002
KX302551	02KE411gag.M1	411	M1	gag	2002
KX302552	02KE411gag.M3	411	M3	gag	2002
KX302553	02KE411gag.M6	411	M6	gag	2002
KX302554	02KE411gag.M9	411	M9	gag	2002
KX302555	02KE411nef.M1	411	M1	nef	2002
KX302556	02KE411nef.M3	411	M3	nef	2002
KX302557	02KE411nef.M6	411	M6	nef	2002
KX302558	02KE411nef.M9	411	M9	nef	2002
KX302559	02KE411pol.M1	411	M1	pol	2002
KX302560	02KE411pol.M3	411	M3	pol	2002
KX302561	02KE411pol.M6	411	M6	pol	2002
KX302562	02KE411pol.M9	411	M9	pol	2002
KX302563	02KE424gag.M1	424	M1	gag	2002
KX302564	02KE424gag.M2.5	424	M2	gag	2002
KX302565	02KE424gag.M6	424	M6	gag	2002
KX302566	02KE424nef.M1	424	M1	nef	2002
KX302567	02KE424nef.M2.5	424	M2	nef	2002
KX302568	02KE424nef.M6	424	M6	nef	2002
KX302569	02KE440gag.M1	440	M1	gag	2002
KX302570	02KE440gag.M6	440	M6	gag	2002
KX302571	02KE440gag.NEO	440	NEO	gag	2002
KX302572	02KE440nef.M1	440	M1	nef	2002
KX302573	02KE440nef.M6	440	M6	nef	2002
KX302574	02KE454gag.M1	454	M1	gag	2002
KX302575	02KE454gag.M3	454	M3	gag	2002
KX302576	02KE454nef.M1	454	M1	nef	2002
KX302577	02KE454nef.M3	454	M3	nef	2002
KX302578	02KE454pol.M1	454	M1	pol	2002
KX302579	02KE454pol.M3	454	M3	pol	2002
KX302580	02KE485gag.M3	485	M3	gag	2002
KX302581	02KE485gag.NEO	485	NEO	gag	2002
KX302582	02KE485pol.M3	485	M3	pol	2002

KX302583	02KE485pol.NEO	485	NEO	pol	2002
KX302584	03KE440gag.M9	440	M9	gag	2003
KX302585	03KE440nef.M9	440	M9	nef	2003
KX302586	03KE454gag.M15	454	M15	gag	2003
KX302587	03KE454gag.M6	454	M6	gag	2003
KX302588	03KE454nef.M15	454	M15	nef	2003
KX302589	03KE454nef.M6	454	M6	nef	2003
KX302590	03KE454pol.M15	454	M15	pol	2003
KX302591	03KE454pol.M6	454	M6	pol	2003
KX302592	03KE485gag.M6	485	M6	gag	2003
KX302593	03KE485gag.M9	485	M9	gag	2003
KX302594	03KE485pol.M6	485	M6	pol	2003

M, month.

Supplementary References

1. de Silva TI, et al. (2010) HIV-1 subtype distribution in the Gambia and the significant presence of CRF49_cpx, a novel circulating recombinant form. *Retrovirology* 7(1):82.

2. HIV Databases HIV sequence database (accessed Spetember 2013). Available at: http://www.hiv.lanl.gov/content/sequence/HIV/mainpage.html.
